# 1012. Outcomes of a Quality Improvement Initiative: Multi-Disciplinary Team for Infective Endocarditis Improves the Numbers of Patients Who Undergo Surgery

**DOI:** 10.1093/ofid/ofac492.853

**Published:** 2022-12-15

**Authors:** Haley W Crosby, Robert P Pierce, Hariharan Regunath

**Affiliations:** University of Missouri, Columbia, Missouri; University of Missouri, Columbia, Missouri; University of Missouri, Columbia, Missouri

## Abstract

**Background:**

In a prior study we identified leverage points for improving infective endocarditis (IE) outcomes at an academic medical center¹. We aimed to improve the rate of surgery for those with guideline-based indications for surgery by 50%.

**Methods:**

We recorded outcomes and surgical indications for patients with IE from December 2018 to June 2020 and compared to our prior published data from January to December 2016, using similar criteria.¹ Changes implemented in the interim period included development of a multidisciplinary team (MDT) for IE that provided recurring conferences, participated in heart valve team case discussions, and promoted the use of a home-grown algorithmic clinical care pathway within the electronic health record to guide providers on the next steps in management. Primary outcomes were surgery or transfer to a higher center for surgery, and in-hospital death. Odds ratios were calculated using a multivariate logistic regression model including age and sex covariates.

**Results:**

We identified 31 IE patients with guideline indications for surgery. Of those patients, 15(48.39%) were female, 15(48.4%) were 18 - 44 years of age, 8(25.8%) were 45 - 64 years, 8(25.8%) were >64 years, 28(90.3%) white, 2(6.4%) black, 1(3.2%) East Asian, 17(54.8%) were intravenous drug users. Prior to the intervention, 6 of 21 (28.6%) patients with indications underwent surgery or were transferred outside for surgery and 6 (28.6%) patients died. Post-intervention, 17 of 31 (54.8%) patients with indications underwent or were transferred for surgery, and 5 (16.1%) died. After adjustment for age and sex, compared to the pre-intervention period, the odds of surgery or transfer for surgery for patients in the post-intervention period was 4.88 (95% CI 1.20, 19.79, p=.027). The odds ratio for death among patients in the post-intervention period was 0.40 (95% CI 0.09, 1.69, p=0.21).

Pre- and post-intervention outcomes for infective endocarditis patients

**Figure:**
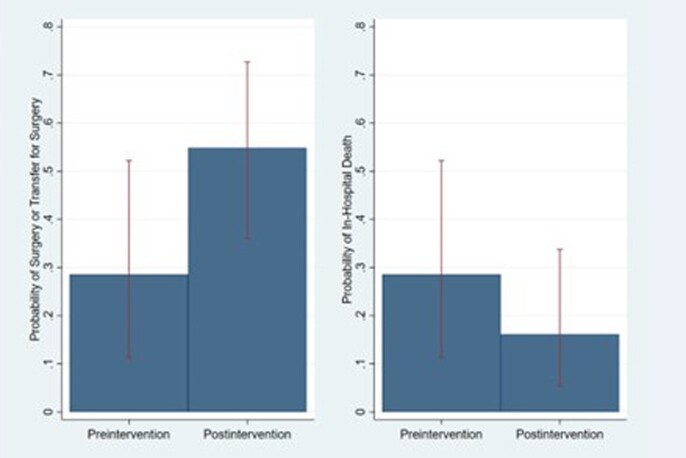

**Conclusion:**

MDT team with continued educational and health IT interventions improved the number of surgeries performed for IE.

1. Regunath H, Vasudevan A, Vyas K, et al. *A Quality Improvement Initiative:* Developing a Multi-Disciplinary Team for Infective Endocarditis. *Mo Med*. 2019;116(4):291-296.

**Disclosures:**

**All Authors**: No reported disclosures.

